# Intestinal subepithelial myofibroblasts in inflammatory bowel disease: fibroblast heterogeneity, fibrosis, and therapeutic targeting

**DOI:** 10.3389/fmed.2026.1808431

**Published:** 2026-06-24

**Authors:** Fang Li, Chao Xu, Jun Chen, Teng Lei, Xigui Tian, Yuanling Zhang, Guoqing Chen

**Affiliations:** Department of General Surgery, Chongqing General Hospital, Chongqing, China

**Keywords:** antifibrotic therapy, immune–stromal crosstalk, inflammatory bowel disease, intestinal fibrosis, intestinal subepithelial myofibroblasts

## Abstract

Intestinal fibrosis remains one of the most challenging complications of inflammatory bowel disease (IBD), frequently leading to bowel strictures, impaired intestinal function, and surgical intervention. Among the stromal cell populations involved in intestinal remodeling, intestinal subepithelial myofibroblasts (ISEMFs) have emerged as central regulators of epithelial homeostasis, mucosal repair, immune responses, and extracellular matrix remodeling. However, persistent inflammatory and microbial stimuli can drive pathological ISEMF activation, resulting in excessive matrix deposition and progressive fibrosis. This review aims to provide a comprehensive overview of the physiological and pathological roles of ISEMFs in IBD. We first discuss their functions in maintaining epithelial integrity, supporting the intestinal stem-cell niche, and coordinating tissue repair. We then examine the mechanisms underlying ISEMF activation during chronic inflammation, focusing on immune–stromal crosstalk, profibrotic cytokines, mechanotransduction, metabolic reprogramming, and key signaling pathways including TGF-β/Smad, JAK/STAT, and Wnt/β-catenin. Particular attention is given to recent advances in single-cell and spatial transcriptomics that have revealed marked fibroblast heterogeneity and identified pathogenic fibroblast subsets associated with fibrostenotic disease. Finally, we summarize current and emerging therapeutic strategies targeting stromal pathways, including antifibrotic agents, cytokine-directed therapies, fibroblast subset–specific interventions, and biomarker-guided precision approaches. By integrating advances in stromal biology, fibrosis mechanisms, and translational therapeutics, this review highlights ISEMFs as key drivers of intestinal fibrosis and promising targets for future disease-modifying therapies in IBD.

## 1 Introduction

Inflammatory bowel diseases (IBD), including Crohn’s disease (CD) and ulcerative colitis (UC), are chronic relapsing inflammatory disorders caused by dysregulated immune responses to intestinal microbiota in genetically susceptible individuals (1, 2). Despite major advances in biologic and small-molecule therapies that effectively suppress inflammation, a substantial proportion of patients develop progressive intestinal fibrosis, a complication that remains largely refractory to current medical treatment (3, 4). Fibrosis represents a maladaptive wound-healing response characterized by excessive extracellular matrix (ECM) deposition, resulting in bowel wall stiffening, luminal narrowing, stricture formation, and intestinal obstruction (5).

Among the mesenchymal cell populations implicated in intestinal remodeling, intestinal subepithelial myofibroblasts (ISEMFs) have emerged as central cellular mediators linking chronic inflammation to fibrogenesis (6, 7). ISEMFs are spindle-shaped stromal cells located immediately beneath the intestinal epithelium, forming the pericryptal fibroblast sheath and expressing mesenchymal markers such as vimentin, with inducible expression of α-smooth muscle actin (α-SMA) during activation (6, 7). Under physiological conditions, ISEMFs contribute to mucosal homeostasis by regulating ECM turnover, producing growth factors and cytokines that support epithelial renewal, modulating immune cell behavior, and coordinating tissue repair after injury (7, 8).

ISEMFs play a key role in epithelial regeneration and wound healing. During acute mucosal injury, these cells proliferate and migrate to sites of damage, where they secrete provisional matrix components and growth factors that promote epithelial restitution (7). *In vitro* studies demonstrate that subepithelial myofibroblasts enhance epithelial proliferation and differentiation through paracrine signaling, while also supporting angiogenesis by interacting with endothelial cells in three-dimensional culture systems (7, 8). These functions highlight the essential role of ISEMFs in coordinated epithelial–mesenchymal interactions during normal tissue repair.

In the setting of chronic intestinal inflammation, however, ISEMFs undergo persistent pathological activation, acquiring a myofibroblast phenotype characterized by enhanced proliferation, migratory capacity, and excessive synthesis of ECM proteins such as collagen I, collagen III, fibronectin, and laminin (3, 8). This activation is driven by the inflammatory microenvironment of IBD lesions. Cytokines including TNF-α, IL-1β, IL-6, IL-17A, and IL-13 stimulate ISEMFs to produce profibrotic mediators such as TGF-β1 and connective tissue growth factor. These responses sustain a vicious cycle of inflammation and fibrosis (3).

A growing body of evidence indicates that intestinal fibroblasts and related mesenchymal cells are highly heterogeneous rather than functionally uniform. Single-cell and spatial transcriptomic studies have identified anatomically localized and transcriptionally distinct fibroblast programs linked to epithelial support, immune regulation, tissue repair, and profibrotic remodeling (9, 10). This emerging view of stromal diversity is particularly relevant to IBD, in which specific fibroblast states appear to contribute disproportionately to chronic inflammation, matrix deposition, and fibrostenotic complications (9).

Unlike physiological wound healing, in which myofibroblasts are transient and undergo apoptosis after tissue repair, ISEMF activation in IBD becomes persistent, leading to progressive collagen accumulation and irreversible scarring of the bowel wall (4, 5). Importantly, although inflammation is typically required to initiate fibrogenic responses, experimental and clinical observations suggest that established intestinal fibrosis may not fully regress after inflammatory activity is controlled. Persistent matrix deposition, increased tissue stiffness, apoptosis-resistant myofibroblasts, and mechanotransduction-dependent activation of latent TGF-β can create feed-forward loops that maintain stromal activation. However, direct evidence demonstrating truly inflammation-independent progression in intestinal fibrosis models remains limited, and this concept should be interpreted as partial persistence of fibrotic remodeling rather than complete autonomy from inflammation (11). Notably, intestinal fibrosis often progresses independently of active inflammation, explaining why strictures may develop or worsen even in patients who achieve clinical or endoscopic remission (3). Clinically, more than half of patients with Crohn’s disease develop fibrostenotic complications within 20 years of diagnosis, frequently necessitating endoscopic dilation or surgical resection despite optimized medical therapy (12–14).

**Figure 1:**
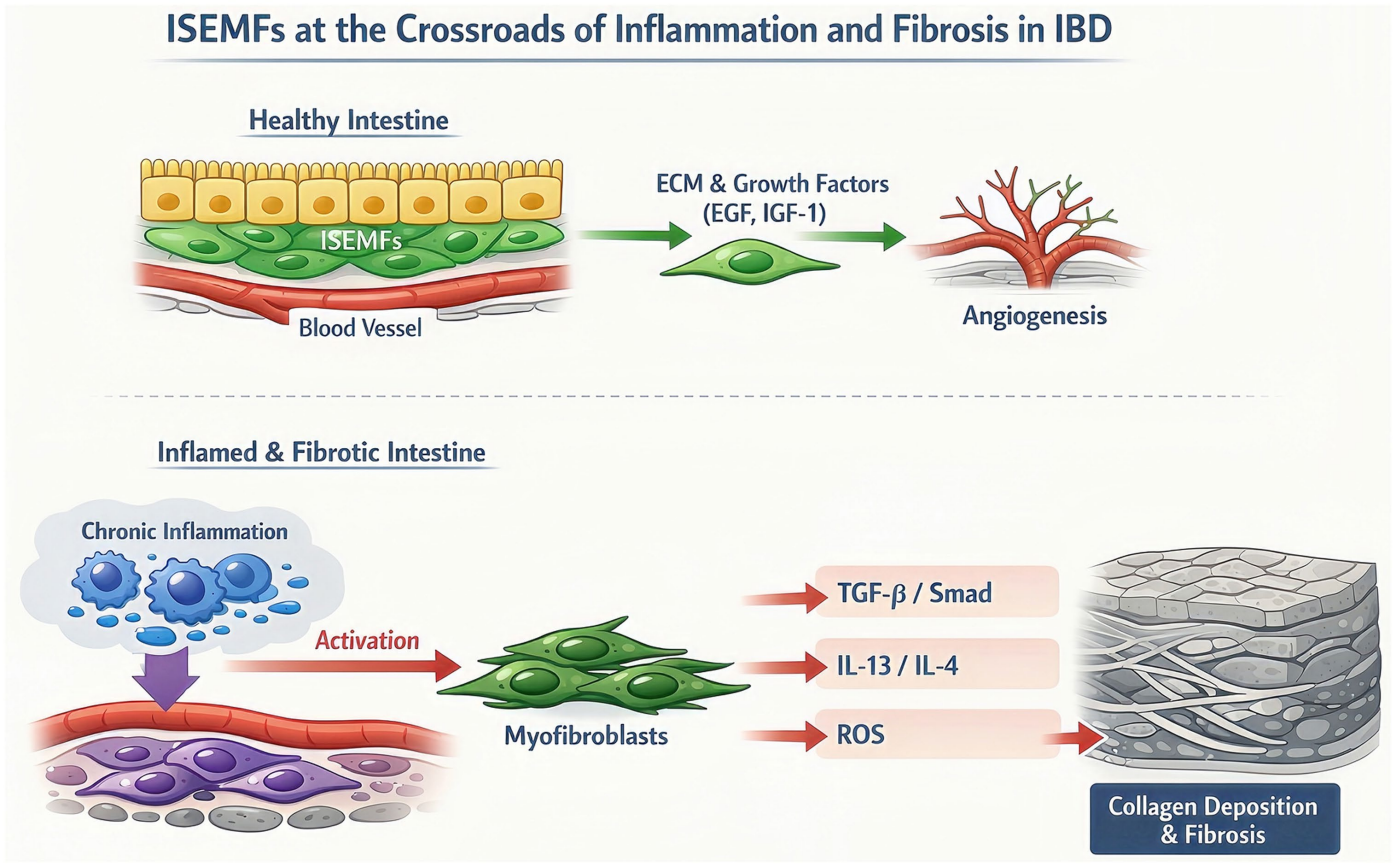
Intestinal subepithelial myofibroblasts at the intersection of mucosal repair and fibrosis in inflammatory bowel disease. In the healthy intestine (left), intestinal subepithelial myofibroblasts (ISEMFs; green) reside beneath the epithelium (yellow) and support mucosal homeostasis by producing structural extracellular matrix (ECM), epithelial growth factors (e.g., EGF, IGF-1), and pro-angiogenic signals that sustain the microvasculature (red). Following acute injury, transient ISEMF activation facilitates epithelial restitution and tissue regeneration. In inflammatory bowel disease (IBD; right), persistent immune activation (immune cells shown in blue) drives pathological ISEMF differentiation into contractile, collagen-producing myofibroblasts (dark green). These activated cells secrete excessive ECM components, pro-fibrotic cytokines, and chemokines, resulting in submucosal thickening and fibrotic remodeling (gray). Key profibrotic pathways upregulated in activated ISEMFs include TGF-β/Smad signaling, IL-13/IL-4–driven responses from type 2 immunity, macrophage-derived mediators, and reactive oxygen species (ROS), collectively promoting collagen deposition, tissue stiffening, and stricture formation.

In this review, we examine ISEMF biology across homeostasis, inflammation, repair, and fibrosis, with particular emphasis on fibroblast heterogeneity, immune–stromal crosstalk, and profibrotic signaling pathways. We also discuss experimental models and therapeutic strategies aimed at modulating pathogenic stromal responses while preserving regenerative functions. As illustrated in Figure 1, intestinal subepithelial myofibroblasts occupy a central position at the interface of epithelial repair, immune activation, and fibrotic remodeling in inflammatory bowel disease, acting as key determinants of whether inflammation resolves with regeneration or progresses to irreversible fibrosis.

## 2 Physiology of intestinal subepithelial myofibroblasts

### 2.1 Characteristics of intestinal subepithelial myofibroblasts

Intestinal subepithelial myofibroblasts (ISEMFs) are specialized mesenchymal cells exhibiting a hybrid phenotype between fibroblasts and smooth muscle cells. Morphologically, they are elongated, spindle-shaped, contractile cells located immediately beneath the intestinal epithelium, particularly at the base of the crypts and along the villus core, where they are closely apposed to epithelial cells, endothelial cells, and pericytes (6, 7). This strategic localization enables ISEMFs to function as a structural and signaling interface between the epithelium and the lamina propria.

Intestinal subepithelial myofibroblasts (ISEMFs) are classically defined as mesenchymal cells located immediately beneath the intestinal epithelium, where they contribute to epithelial support, extracellular matrix turnover, and wound repair (6, 7). However, recent single-cell transcriptomic and spatial studies indicate that the subepithelial mesenchymal compartment is not uniform and includes several related but distinct stromal populations, including telocytes, broader PDGFRA^+^ stromal cells, trophocyte-like fibroblasts, and perivascular pericytes (15, 16). In this review, we use the term ISEMFs primarily in the anatomical and functional sense to denote subepithelial stromal/myofibroblast populations directly apposed to the epithelium, while acknowledging that not all cells within this compartment are identical at the molecular level.

ISEMFs express classical mesenchymal markers, including vimentin and fibroblast-specific protein-1 (FSP1/S100A4), and upregulate α-smooth muscle actin (α-SMA) upon activation, conferring contractile properties that distinguish them from quiescent fibroblasts (6, 17). In their resting state, ISEMFs typically express low or undetectable levels of α-SMA and lack desmin expression, a feature that differentiates them from true smooth muscle cells of the muscularis mucosae and muscularis propria (7). Functionally, ISEMFs are major producers of extracellular matrix components, including collagen types I and III, fibronectin, and laminin, which together form the structural scaffold of the intestinal mucosa (3). Through continuous synthesis and remodeling of basement membrane and interstitial matrix components, ISEMFs help maintain crypt architecture, epithelial anchorage, and tissue tensile strength.

Beyond their structural role, ISEMFs are increasingly recognized as critical regulators of the intestinal epithelial stem cell niche. They reside immediately beneath crypt base columnar cells, where Lgr5^+^ intestinal stem cells are located, and provide paracrine signals that support epithelial renewal (7). Among these signals, Wnt pathway activity is essential for stem cell maintenance. Although multiple mesenchymal populations contribute to Wnt signaling *in vivo*, subepithelial myofibroblasts have been shown to secrete Wnt ligands and Wnt-modulating factors that promote epithelial proliferation and differentiation (7, 18). *In vitro* co-culture experiments demonstrate that intestinal epithelial cells grown in the presence of ISEMFs or their conditioned media exhibit enhanced proliferation, survival, and regenerative capacity compared with epithelial monocultures, underscoring the importance of epithelial–mesenchymal crosstalk in mucosal homeostasis (7).

ISEMFs also contribute to mucosal repair by producing growth factors in response to injury. Following epithelial damage, ISEMFs upregulate epidermal growth factor (EGF) and insulin-like growth factor-1 (IGF-1), which stimulate epithelial cell proliferation, migration, and restitution, thereby facilitating re-establishment of barrier integrity (3). In parallel, ISEMFs express vascular endothelial growth factor (VEGF) and other pro-angiogenic mediators that support microvascular remodeling during tissue repair. Experimental studies have shown that intestinal myofibroblasts promote endothelial cell organization and capillary-like structure formation in three-dimensional culture systems, highlighting their role in coordinating epithelial repair with angiogenesis (19).

FOXL1^+^ telocytes have been identified as a key component of the intestinal stem cell niche and an important source of Wnt ligands required for epithelial renewal, with spatial compartmentalization of both Wnt agonists and inhibitors along the crypt–villus axis (20, 21). By contrast, PDGFRA^+^ stromal cells represent a broader mesenchymal category that includes multiple fibroblast-like subsets, some of which overlap with telocyte-like or subepithelial populations, whereas pericytes are primarily perivascular mesenchymal cells associated with the microvasculature and should not be conflated with subepithelial myofibroblasts (15, 22).

### 2.2 Role of ISEMFs in intestinal homeostasis

ISEMFs function as key “gatekeepers” of the mucosal microenvironment, dynamically responding to injury signals while maintaining epithelial and immune homeostasis. During normal wound repair, acute epithelial injury triggers ISEMF proliferation and migration toward the wound bed, where they transiently differentiate into activated myofibroblasts. These cells generate contractile forces that facilitate wound closure and deposit a provisional extracellular matrix that supports epithelial restitution (3). As healing progresses, myofibroblasts undergo apoptosis or revert to a quiescent phenotype, while the provisional matrix is remodeled by matrix metalloproteinases, preventing excessive scar formation (4). In this resolution phase, ISEMFs express matrix metalloproteinases such as MMP-3 and MMP-9, which contribute to controlled collagen degradation and tissue remodeling (8).

ISEMFs also participate in terminating inflammation once tissue repair is underway. They produce anti-inflammatory mediators, including prostaglandin E₂ and transforming growth factor-β (TGF-β), which help suppress ongoing immune activation and restore mucosal homeostasis (3, 14). Through these mechanisms, ISEMFs couple the resolution of inflammation to the restoration of tissue architecture.

In the steady state, ISEMFs contribute to maintenance of the epithelial barrier by providing structural support to crypt and villus units and by regulating paracrine signaling to epithelial cells. Experimental evidence suggests that ISEMF-derived mediators such as nitric oxide and prostaglandins can influence epithelial tight junction integrity, mucus secretion, and epithelial differentiation, thereby reinforcing barrier function (7). In addition, ISEMFs exhibit features of non-professional antigen-presenting cells: they express major histocompatibility complex class II molecules and can present antigen to CD4^+^ T cells under inflammatory conditions, albeit less efficiently than professional antigen-presenting cells such as dendritic cells (6, 7). This capacity suggests a potential role for ISEMFs in shaping local immune responses at the epithelial–stromal interface.

ISEMFs engage in extensive bidirectional crosstalk with immune cells in the lamina propria. Under homeostatic conditions, they contribute to immune tolerance by responding to anti-inflammatory cytokines such as interleukin-10 and by supporting regulatory immune circuits (3). During inflammation, however, exposure to Th1- and Th17-associated cytokines—including interferon-*γ* and interleukin-17A—shifts ISEMFs toward a pro-inflammatory phenotype. Activated ISEMFs secrete cytokines and chemokines such as interleukin-6, interleukin-8, and CCL20, which promote recruitment and expansion of inflammatory T cells and neutrophils, thereby amplifying mucosal inflammation (8, 14). Thus, depending on the cytokine milieu, ISEMFs can either restrain or propagate intestinal immune responses.

An emerging aspect of ISEMF physiology is its interaction with the gut microbiome. Microbial products and metabolites can directly influence stromal cell behavior. For example, bacterial lipopolysaccharide can activate ISEMFs via Toll-like receptor 4, inducing NF-κB signaling and pro-inflammatory cytokine production, thereby linking microbial dysbiosis to stromal activation (3). Conversely, microbiota-derived metabolites such as indole-3-propionic acid, a tryptophan catabolite, have been shown to engage the pregnane X receptor in intestinal stromal cells and attenuate inflammatory and fibrotic responses in experimental models (23). These findings highlight how diet–microbiome–stromal interactions can shape ISEMF function and influence the balance between regenerative healing and pathological remodeling.

They also respond to microbiota-derived metabolites, including indole-3-propionic acid (IPA). In DSS-based mouse models of colitis-associated repair/fibrosis, fibroblast-specific PXR (Nr1i2) deficiency, primary intestinal myofibroblast culture experiments, and human IBD biopsy analyses, IPA–PXR signaling was shown to restrain inflammatory activation and fibrotic remodeling, supporting a role for microbiota-dependent stromal regulation in mucosal healing (24).

Furthermore, ISEMFs participate in host–microbiota interactions that influence barrier repair and fibrotic remodeling. They respond to microbiota-derived metabolites, including indole-3-propionic acid (IPA), through pathways such as pregnane X receptor (PXR) signaling. In DSS-induced mouse models, together with fibroblast-specific and epithelial-specific Nr1i2 knockout systems, primary intestinal myofibroblast stimulation assays, and human IBD biopsy analyses, IPA–PXR signaling was shown to suppress inflammatory mediator production and limit fibrosis, indicating that microbial metabolites can directly shape stromal repair programs (24). Disruption of this axis may impair mucosal healing and favor chronic inflammation or fibrosis.

In summary, ISEMFs are multifunctional mesenchymal cells that play indispensable roles in epithelial renewal, immune regulation, and tissue repair under physiological conditions. When chronically exposed to inflammatory and microbial stimuli, however, these same cells can be reprogrammed into persistent drivers of fibrosis. Understanding the mechanisms governing this functional switch is essential for developing therapies that preserve normal repair while preventing pathological tissue remodeling.

As summarized in Figure 1, ISEMFs act as central regulators of intestinal homeostasis by coordinating epithelial repair, extracellular matrix turnover, and vascular support under physiological conditions. In IBD, however, chronic immune stimulation reprograms ISEMFs toward a persistently activated, fibrogenic phenotype that sustains inflammation and drives progressive intestinal fibrosis.

Collectively, intestinal subepithelial myofibroblasts orchestrate epithelial repair, immune modulation, extracellular matrix remodeling, and vascular homeostasis under physiological conditions. In inflammatory bowel disease, however, these tightly regulated functions become dysregulated, shifting ISEMFs toward persistent activation and fibrogenesis. The contrasting roles of ISEMFs in intestinal homeostasis versus chronic inflammation are summarized in Table **1**.

**Table 1:** Functional comparison of intestinal subepithelial myofibroblasts (ISEMFs) in normal physiology and inflammatory bowel disease.

Functional domain	Homeostasis (healthy gut)	IBD (chronic inflammation)	Key mediators/ pathways	Representative evidence (examples)	Translational implication	Reference
Tissue repair & epithelial restitution	Coordinate rapid restitution after injury by remodeling provisional ECM and providing paracrine cues that support re-epithelialization and barrier reconstitution.	Persistent activation → prolonged matrix deposition, impaired “switch-off,” and maladaptive repair favoring scarring/stricture.	TGF-β (dose/context dependent), EGFR ligands, wound-healing cytokines; balance of MMPs/TIMPs.	Chronic stromal activation programs in IBD are enriched for injury-response modules and correlate with refractory disease behavior.	Therapeutic goal: promote “quality healing” (restore repair programs while ensuring myofibroblast resolution).	Shoshkes-Carmel et al. (21)
ECM production and mechanical remodeling	Basal production of collagen/laminin/fibronectin supports crypt–villus architecture; controlled remodeling maintains compliance.	Excess collagen I/III and fibronectin → stiff ECM; mechanotransduction sustains fibrogenic state even when inflammation wanes.	TGF-β/SMAD; integrin–FAK; YAP/TAZ mechanosensing; TIMP-biased remodeling.	Fibrosis can become partially autonomous once ECM stiffness and stromal activation loops are established (concept supported by human stricturing biology).	Combine anti-inflammatory therapy with anti-fibrotic/mechanomodulatory strategies.	West et al. (39)
Stem-cell niche support & epithelial–mesenchymal signaling	Subepithelial stromal networks provide essential niche ligands (WNTs and related cues) that sustain crypt stem cell renewal and epithelial turnover.	Dysregulated niche signaling may skew toward aberrant regeneration, maladaptive remodeling, and epithelial plasticity programs under chronic cytokine stress.	WNT ligands; stromal niche factors; injury-induced epithelial–stromal feedback.	FOXL1 + subepithelial telocytes are a key WNT source required for crypt maintenance.	Targeting “pathologic niche” signals while sparing homeostatic niche is central to precision anti-fibrotic design.	Venkatesh et al. (23)
Immune modulation & tolerance	Stromal cells help maintain immune homeostasis by shaping local cytokine tone, supporting regulatory programs, and limiting excessive leukocyte recruitment.	Shift to pro-inflammatory stromal phenotype → chemokine secretion that recruits/retains myeloid and T cells, fueling chronicity.	Chemokines (CCL2, CXCL8/IL-8, CXCL9/10/11), adhesion molecules (VCAM-1), NF-κB/JAK–STAT.	An OSM-driven stromal inflammatory program predicts anti-TNF non-response; OSM potently activates stromal cells and amplifies leukocyte-recruiting chemokines.	In anti-TNF–refractory IBD, stromal-directed approaches (e.g., OSM–OSMR axis, JAK/STAT) may be needed.	Danese et al. (66)
Cytokine integration hub (immune → stroma)	Respond to acute cytokine cues to coordinate repair, then return toward quiescence.	Continuous exposure to TNF, IL-1β, IL-6-family cytokines and IL-17/IL-23–driven inflammation → persistent activation and fibrogenesis.	TNF; IL-17A; IL-6-family cytokines (OSM/IL-11); TGF-β; STAT3.	Anti-TNF antibodies can directly modulate Crohn’s disease myofibroblasts (migration, MMP/TIMP balance, apoptosis susceptibility), supporting a direct stromal component of response.	Supports rationale for dual-action regimens: immune suppression plus direct stromal/fibrosis modulation.	Di Sabatino et al. (93)
Microbiome–metabolite sensing	Microbial metabolites support barrier and homeostatic tone; stromal/epithelial sensors integrate metabolite signals to restrain inflammation.	Dysbiosis may reduce protective metabolites and increase microbial ligands that activate stromal inflammatory signaling.	PXR signaling; TLR pathways; metabolite-regulated barrier genes.	Microbial indole metabolite indole-3-propionic acid signals via PXR (with TLR4 interplay) to strengthen barrier and suppress inflammatory tone.	Diet/microbiome interventions could be adjunctive to keep stroma in a reparative, non-fibrogenic state.	Sandborn et al. (94)
Angiogenesis and vascular remodeling	Promote physiologic angiogenesis during healing and maintain mucosal microvasculature integrity.	Pathologic angiogenesis → leaky, tortuous vessels that sustain leukocyte trafficking and chronic inflammation; may also contribute to vascular changes within strictures.	VEGF and angiogenic mediators; inflammatory angiogenesis coupling.	Angiogenesis is mechanistically intertwined with chronic intestinal inflammation and may perpetuate disease.	Anti-angiogenic modulation must balance healing vs. inflammatory neovascularization.	Loftus et al. (95)

This table summarizes the principal homeostatic and pathogenic functions of ISEMFs. It contrasts their roles in epithelial support, tissue repair, and immune regulation under physiological conditions with their contributions to chronic inflammation, chemokine production, and fibrotic remodeling in IBD.

## 3 ISEMFs in IBD: inflammation and fibrosis mechanisms

### 3.1 Pathophysiology of IBD: inflammation as a driver of fibrosis

In this section, we focus first on mechanisms that have been demonstrated directly in intestinal ISEMFs or intestinal myofibroblasts, and then briefly note profibrotic mechanisms inferred from other organs that are likely relevant to intestinal fibrosis but remain less well validated in gut stromal cells.

Inflammatory bowel disease is characterized by chronic, immune-mediated tissue injury resulting from a dysregulated interaction between host immunity, microbiota, and genetic susceptibility. In Crohn’s disease (CD), inflammation is typically transmural and may involve all layers of the bowel wall, whereas ulcerative colitis (UC) is largely confined to the mucosa and submucosa of the colon (25, 26). Persistent inflammation in both conditions is sustained by complex cytokine networks, including tumor necrosis factor-α (TNF-α), interleukin-1β (IL-1β), IL-6, IL-21, and the IL-23/IL-17 axis, which together promote continuous immune cell recruitment and tissue damage.

Chronic exposure to this inflammatory milieu initiates and perpetuates intestinal fibrogenesis by directly activating mesenchymal cells, including intestinal subepithelial myofibroblasts (ISEMFs). Pro-inflammatory cytokines such as TNF-α and IL-17A not only exacerbate epithelial injury but also act directly on intestinal fibroblasts to induce profibrotic programs. Experimental studies using human intestinal myofibroblasts have shown that combined TNF-α and IL-17A stimulation synergistically induces transforming growth factor-β1 (TGF-β1) expression and upregulates tissue inhibitor of metalloproteinases-1 (TIMP-1), thereby suppressing extracellular matrix (ECM) degradation and favoring collagen accumulation (27). This shift in matrix homeostasis represents a critical early step in fibrosis development.

Chronic inflammation drives fibrosis through both direct stromal activation and indirect remodeling of the tissue microenvironment. In intestinal myofibroblasts, several profibrotic mechanisms have been demonstrated directly. TNF-α increases collagen accumulation and proliferation in intestinal myofibroblasts, while OSM induces a pro-inflammatory stromal phenotype characterized by increased expression of chemokines such as CCL2, CXCL9, and CXCL10, thereby enhancing immune-cell recruitment and persistence of inflammation (28, 29). Human Crohn’s disease studies also support a role for TGF-β-associated signaling and matrix-remodeling imbalance in stricturing lesions (30).

In parallel, inflammation reshapes the extracellular matrix, increasing tissue stiffness and creating conditions that favor persistent fibrogenic activation. Mechanotransduction is therefore likely to amplify intestinal fibrosis, although some of its best-defined molecular circuits have been established in lung, liver, and skin fibrosis rather than directly in intestinal ISEMFs. A particularly important example is integrin αv–mediated activation of latent TGF-β, which provides a mechanistic bridge between matrix tension and profibrotic signaling. Intestinal studies support the relevance of this axis: αvβ3-dependent activation of latent TGF-β1 has been linked to Crohn’s disease and experimental colitis, and more recent work shows that integrin αvβ6 promotes intestinal fibrosis through FAK/AKT signaling (31, 32). Together, these findings suggest that inflammatory injury, matrix stiffening, αv-integrin signaling, and TGF-β activation form a feed-forward loop that helps sustain fibrogenesis.

Fibrosis is most prominent in Crohn’s disease, where up to 50% of patients develop fibrostenotic complications within the first decade of disease (33). The transmural nature of CD inflammation, involvement of the muscularis propria, and expansion of mesenteric fat (“creeping fat”) create a microenvironment particularly conducive to fibrosis. In contrast, ulcerative colitis rarely leads to clinically significant strictures, likely reflecting its predominantly superficial inflammatory pattern (34). Regional differences also exist within CD; ileal disease is more prone to fibrosis than colonic disease, potentially due to intrinsic differences in stromal cell populations or microbial exposure.

### 3.2 ISEMF activation and immune crosstalk in IBD

Recent single-cell RNA-seq and spatial transcriptomic studies indicate that immune–stromal crosstalk in IBD is not uniform, but instead occurs through anatomically organized fibroblast subsets and their preferred immune partners in distinct inflammatory niches.

In human ileal Crohn’s disease, emerging data suggest that fibroblast-derived mediators such as CCL2 contribute to communication with myeloid cells, and recent work in fibrostenotic disease supports pathogenic interaction between FAP^+^ fibroblasts and inflammatory monocyte/macrophage-lineage populations (35, 36). Together, these studies strengthen the view that fibroblast subsets differ in their immune-interacting programs, with some clusters enriched for chemokines and others more closely linked to matrix remodeling or fibrotic progression.

Some of these interactions are demonstrated directly in human tissue, whereas others are better resolved in mouse models. For example, experimental colitis studies have mapped inflammation-associated fibroblast states in defined tissue neighborhoods and linked them to stage-specific immune interactions, with analogous signatures observed in human ulcerative colitis, suggesting partial conservation across species (37).

In the inflamed IBD mucosa, ISEMFs are activated by a broad array of immune-derived signals, reflecting intense bidirectional crosstalk between stromal and immune compartments. Among the most potent profibrotic mediators are type 2 cytokines, particularly interleukin-13 (IL-13). IL-13 is elevated in ulcerative colitis and fibrotic Crohn’s disease and directly induces intestinal fibroblast differentiation into α-SMA–expressing myofibroblasts while enhancing collagen synthesis through STAT6-dependent pathways (38). Neutralization of IL-13 in experimental colitis models reduces collagen deposition, supporting its role in intestinal fibrosis.

Macrophages are central orchestrators of this fibrogenic environment. Alternatively activated (M2-like) macrophages, polarized by IL-4 and IL-13, accumulate in fibrotic intestinal tissue and secrete TGF-β, platelet-derived growth factor (PDGF), and other growth factors that stimulate ISEMF proliferation and matrix production (38, 39). In human Crohn’s disease strictures, macrophages frequently localize adjacent to α-SMA^+^ fibroblast clusters, highlighting their close spatial and functional interaction.

Th17-associated cytokines also contribute to immune–stromal crosstalk. IL-17A directly stimulates intestinal myofibroblasts to produce IL-6, chemokines, and matrix components, thereby amplifying inflammatory and fibrogenic circuits (27). Oncostatin M (OSM), a cytokine produced by activated T cells and macrophages, has emerged as a key mediator linking inflammation to stromal activation. OSM signaling in intestinal fibroblasts induces expression of chemokines (e.g., CCL2, CXCL9, CXCL10) and adhesion molecules such as VCAM-1, converting ISEMFs into potent recruiters of inflammatory leukocytes (39). A particularly important translational example is the OSM-high stromal inflammatory program. In human IBD mucosal biopsies, West et al. demonstrated that elevated OSM and OSMR expression was associated with stromal activation and predicted non-response to anti-TNF therapy, indicating that stromal cytokine programs may contribute to treatment resistance (39).

Additional immune cell types contribute to fibroblast activation. Mast cells, which are enriched in fibrotic Crohn’s disease segments, release tryptase, histamine, IL-4, and IL-13, all of which promote fibroblast proliferation and myofibroblast differentiation. Dendritic cells, through production of TNF-α, IL-6, and IL-23, further reinforce Th17-driven inflammation and indirectly sustain fibroblast activation.

ISEMFs themselves express pattern-recognition receptors, including Toll-like receptor 4 and NOD-like receptors, enabling them to directly sense microbial products. Engagement of TLR4 by lipopolysaccharide activates NF-κB signaling in intestinal fibroblasts, leading to cytokine and chemokine secretion that shapes local immune responses (40). Genetic variants associated with Crohn’s disease, such as NOD2 polymorphisms, may further modulate fibroblast responses to microbial ligands, although this area remains incompletely defined.

Neutrophil extracellular traps (NETs) represent another emerging link between immune activation and fibrosis. In Crohn’s disease, excessive NET formation has been observed in strictured tissue. NET-associated peptidylarginine deiminases promote citrullination of ECM proteins, enhancing fibroblast activation and collagen production. Experimental inhibition of NET formation or protein citrullination reduces intestinal fibrosis in murine colitis models, implicating NETs as active drivers of stromal activation (41).

### 3.3 Molecular pathways in ISEMF-mediated fibrosis

ISEMF activation in IBD is governed by an interconnected network of molecular pathways that collectively drive fibrogenesis. Central among these is the TGF-β1/Smad pathway, which initiates transcriptional programs responsible for ECM production and myofibroblast differentiation. Intestinal fibroblasts isolated from fibrotic Crohn’s disease tissue display constitutive Smad3 activation and sustained expression of TGF-β target genes, even ex vivo, suggesting stable reprogramming (42).

The IL-6/JAK/STAT3 axis represents another critical profibrotic pathway. IL-6 signaling through STAT3 promotes fibroblast survival, proliferation, and resistance to apoptosis, while also inducing TIMP-1 expression and suppressing ECM degradation (38). Pharmacologic JAK inhibition has been shown to reduce collagen synthesis by intestinal fibroblasts *in vitro*, raising the possibility that JAK inhibitors may exert indirect antifibrotic effects in addition to their anti-inflammatory actions. In addition, some JAK inhibitors already used in IBD treatment may also exert direct antifibrotic effects, although current evidence remains limited and is derived mainly from preclinical or indirect translational studies. Available data suggest that JAK inhibition can modulate fibroblast-associated inflammatory programs and may reduce profibrotic signaling, but definitive antifibrotic effects in intestinal fibrosis have not yet been established clinically (43, 44).

Wnt/β-catenin signaling has recently been implicated in fibroblast activation and persistence. Fibrotic intestinal tissue exhibits increased expression of non-canonical Wnt ligands such as Wnt5a, which promote fibroblast activation and migration. Stromal cell populations enriched for Wnt pathway activation have been identified in strictured Crohn’s disease, suggesting a role for Wnt signaling in maintaining fibrogenic fibroblast subsets (45).

Metabolic reprogramming has emerged as an important feature of activated ISEMFs and other profibrotic fibroblast populations (9, 10). Similar to cancer-associated fibroblasts, activated intestinal myofibroblasts exhibit enhanced aerobic glycolysis, increased glucose uptake, and altered mitochondrial metabolism to support the high biosynthetic demands required for extracellular matrix production. Beyond energy supply, specific metabolites can directly regulate fibrogenic signaling pathways.

Accumulation of succinate, a tricarboxylic acid (TCA) cycle intermediate, has emerged as an important profibrotic signal. Extracellular succinate activates succinate receptor 1 (SUCNR1), promoting inflammatory responses, TGF-β signaling, and fibroblast activation. Although direct evidence in ISEMFs remains limited, studies of intestinal fibrosis suggest that succinate-mediated signaling may contribute to persistent stromal activation and excessive ECM deposition. Likewise, increased lactate production under hypoxic and inflammatory conditions can enhance fibroblast differentiation and collagen synthesis through HIF-1α-dependent pathways, linking tissue hypoxia to fibrotic remodeling (46).

Conversely, several microbiota-derived metabolites appear to exert antifibrotic effects. Short-chain fatty acids (SCFAs), particularly butyrate, can activate AMPK and PPAR-*γ* signaling pathways, thereby suppressing inflammatory and profibrotic responses (11). In addition, tryptophan-derived metabolites such as indole-3-propionic acid (IPA) activate pregnane X receptor (PXR) signaling in intestinal stromal cells and have been shown to limit inflammatory activation and fibrotic remodeling in experimental models (24). Together, these findings suggest that metabolic pathways not only support the energetic demands of activated ISEMFs but also actively regulate stromal phenotypes and fibrosis progression, highlighting metabolic interventions as a promising therapeutic avenue for intestinal fibrosis (11).

Finally, resistance to apoptosis contributes to the persistence of activated ISEMFs. Fibrotic intestinal tissue shows upregulation of anti-apoptotic pathways, including PI3K/Akt and NF-κB signaling, allowing myofibroblasts to evade normal clearance after wound repair. Strategies aimed at selectively inducing apoptosis in activated fibroblasts may therefore help resolve established fibrosis. Preclinical studies have also explored whether reversing apoptosis resistance in intestinal fibroblasts may limit fibrosis. For example, simvastatin attenuated intestinal fibrosis in TNBS-induced colitis while promoting fibroblast/myofibroblast apoptosis, supporting the concept that selective elimination of persistently activated mesenchymal cells may have antifibrotic benefit (47). Earlier work also examined TRAIL-mediated apoptosis in intestinal fibroblasts from Crohn’s disease, although this approach remains experimental (48).

In summary, ISEMF-mediated fibrosis in IBD arises from the convergence of inflammatory, immune, mechanical, and metabolic signals. These pathways reinforce one another, creating a self-sustaining fibrotic program that persists even after inflammation subsides. Effective antifibrotic therapy will likely require combinatorial approaches targeting multiple nodes within this network.

### 3.4 Bone marrow contributions to the fibrogenic ISEMF pool

It is important to note that the evidence for bone marrow-derived fibroblast-like cells and macrophage-to-myofibroblast transition (MMT) is not equally strong across organs. In the intestine, support comes mainly from transplantation-associated regenerative models and indirect lineage inference, whereas more definitive MMT evidence has been established primarily in non-intestinal fibrotic systems (49).

ISEMFs were traditionally considered a resident mesenchymal population within the intestinal mucosa. Evidence for bone marrow contribution to intestinal subepithelial myofibroblast-like populations comes mainly from gut regenerative and inflammatory injury models, including analyses of human sex-mismatched bone marrow transplantation specimens and related experimental observations suggesting that bone marrow-derived cells can populate the colonic subepithelial myofibroblast compartment during mucosal repair (49).

These recruited cells are often referred to as fibrocytes, a population of circulating bone marrow–derived progenitors that express hematopoietic markers (e.g., CD45, CD34) alongside mesenchymal proteins such as collagen I. Fibrocytes are mobilized from the bone marrow during tissue injury and home to inflamed sites in response to chemokines such as CCL2 and CXCL12 (50). Once within the intestinal microenvironment, exposure to profibrotic signals—including TGF-β and IL-13—drives their differentiation into α-SMA–expressing myofibroblasts capable of producing large amounts of extracellular matrix.

In human IBD, increased numbers of circulating fibrocytes have been reported, particularly in patients with stricturing Crohn’s disease, and these cells exhibit enhanced migratory capacity toward inflamed intestinal tissue (51). Histological analyses of fibrotic intestinal segments suggest that bone marrow–derived cells may constitute a subset of the total myofibroblast population, although distinguishing them from resident fibroblasts requires sophisticated lineage-tracing or chimerism approaches.

Recruitment of bone marrow–derived mesenchymal cells appears to represent a physiological attempt to augment tissue repair by supplying additional stromal cells to sites of injury. In the context of chronic inflammation, however, this process becomes maladaptive. Continuous recruitment and differentiation of fibrocytes expand the pool of long-lived myofibroblasts, thereby accelerating fibrosis progression. Experimental blockade of fibrocyte recruitment—such as inhibition of CCR2- or CCR7-mediated trafficking—has been shown to reduce fibroblast accumulation and attenuate fibrosis in models of chronic inflammation (52).

By contrast, MMT in the intestine remains more speculative. Although macrophage–fibroblast interaction is clearly important in intestinal fibrosis, direct demonstration that macrophages convert into matrix-producing myofibroblasts in the gut is still limited, and much of the mechanistic framework is extrapolated from other organs (53). Another proposed mechanism linking bone marrow–derived cells to fibrosis is the macrophage-to-myofibroblast transition (MMT). Much of the current MMT model derives from non-intestinal fibrosis studies, particularly kidney allograft fibrosis, where macrophage-to-myofibroblast transition has been demonstrated using bone marrow-tracking and lineage-associated approaches (54). Similar concepts have been discussed in broader fibrosis reviews and may be relevant to intestinal disease, but direct gut-specific validation remains limited (53).

The involvement of bone marrow–derived cells in intestinal fibrosis has important therapeutic implications. Hematopoietic stem cell transplantation (HSCT) has occasionally induced long-term remission in refractory Crohn’s disease, raising the possibility that resetting both immune and stromal compartments can alter disease trajectory (55). More targeted approaches, such as local delivery of mesenchymal stem cells for fistulizing Crohn’s disease, have demonstrated clinical efficacy in promoting tissue repair (56). While these therapies primarily aim to modulate immunity and enhance healing, their long-term effects on stromal remodeling and fibrosis remain an important area of investigation.

In summary, bone marrow–derived cells represent a dynamic and context-dependent source of ISEMFs during intestinal injury. While they can support mucosal repair, their persistent recruitment and differentiation in chronic IBD contribute to fibrosis evolution. Understanding the signals that govern the balance between regenerative versus fibrogenic differentiation of these cells may enable strategies that harness their reparative potential while limiting their contribution to pathological scarring.

## 4 ISEMFs in regenerative responses and fibrosis evolution

A central physiological role of intestinal subepithelial myofibroblasts (ISEMFs) is to orchestrate the resolution phase of inflammation and promote effective mucosal healing. During acute injury or self-limited inflammation, clearance of pathogens and attenuation of pro-inflammatory signals trigger a functional shift in ISEMFs from a pro-inflammatory, matrix-producing phenotype toward a pro-restitutive state. In this phase, ISEMFs increase production of anti-inflammatory mediators, including interleukin-10 (IL-10), and secrete growth factors that support epithelial restitution and barrier restoration (57).

Transforming growth factor-β1 (TGF-β1) plays a dual role during resolution. At moderate and spatially restricted levels, TGF-β1 contributes to the termination of immune responses, promotes epithelial differentiation, and facilitates the restoration of epithelial barrier integrity (58). ISEMF-derived TGF-β1 also acts on neighboring immune cells, dampening excessive inflammation and coordinating the transition from inflammatory to reparative tissue remodeling. As inflammation subsides, macrophages in the lamina propria undergo a phenotypic switch toward a wound-healing profile characterized by secretion of IL-10 and TGF-β. These macrophage-derived signals act on ISEMFs to promote extracellular matrix reorganization and scar maturation rather than continued matrix expansion (59).

In effective wound healing, ISEMF-driven deposition of provisional extracellular matrix is followed by controlled remodeling. Excess myofibroblasts are eliminated through apoptosis, and collagen is reorganized into a more ordered architecture, restoring tissue compliance. However, in inflammatory bowel disease (IBD), this resolution program is frequently disrupted. Persistent exposure to inflammatory cytokines such as TNF-α and IL-6 rescues ISEMFs from apoptosis and sustains their activated state, allowing continued collagen production even after epithelial healing appears complete (60). Recurrent cycles of inflammation and repair—characteristic of relapsing–remitting IBD—further exacerbate this process, with each inflammatory flare triggering new rounds of matrix deposition before prior scars can be adequately remodeled, ultimately leading to progressive fibrosis.

At present, it is important to distinguish between clinical outcomes reported for microbiome-based interventions and direct mechanistic evidence showing how these interventions regulate stromal or ISEMF-specific repair programs.

The intestinal microbiome also plays a critical role in shaping the resolution response. Commensal-derived metabolites contribute to mucosal healing by influencing stromal cell behavior. Short-chain fatty acids, particularly butyrate, promote epithelial repair and have been shown to modulate fibroblast function toward a reparative phenotype (61). In experimental systems, butyrate enhances production of hepatocyte growth factor (HGF) by intestinal stromal cells, thereby supporting epithelial proliferation and migration during wound repair. Conversely, dysbiosis—characterized by loss of beneficial commensals and expansion of pathobionts—can prolong inflammation and impair ISEMF-mediated resolution by sustaining NF-κB activation and pro-inflammatory cytokine production.

Recent studies have highlighted the role of gut microbiota and diet in modulating inflammation and repair in IBD. However, it is important to distinguish between clinical outcome data and direct mechanistic evidence on stromal repair programs (62). From a clinical perspective, microbiome-based approaches such as fecal microbiota transplantation (FMT) and dietary modulation have shown potential to improve remission rates, mucosal healing, or inflammatory activity in selected IBD settings, particularly in ulcerative colitis, although results remain variable across studies and treatment protocols (63).

By contrast, direct evidence that these interventions specifically reprogram ISEMFs or other intestinal stromal cells toward reparative phenotypes remains relatively sparse. Most mechanistic data are indirect and suggest that microbiota-derived metabolites, epithelial recovery, and altered immune signaling may secondarily shape stromal behavior rather than demonstrating a direct stromal effect *in vivo* (64). Thus, while microbiome-based therapies may create conditions favorable for tissue repair, their ability to drive defined reparative stromal programs in IBD has not yet been clearly established (65).

ISEMFs also coordinate resolution through interactions with other stromal cell populations, particularly endothelial cells and pericytes. During healing, normalization of the microvasculature is essential for restoring tissue oxygenation and nutrient supply. Persistent inflammation disrupts this stromal cross-talk, resulting in abnormal angiogenesis or microvascular distortion that may predispose tissue to chronic injury and fibrosis (66). Advanced endoscopic imaging techniques, including confocal laser endomicroscopy, have revealed subepithelial thickening and altered microvascular patterns in endoscopically healed mucosa, suggesting that incomplete stromal resolution precedes overt fibrotic remodeling (67).

In summary, ISEMFs are indispensable mediators of inflammation resolution and tissue repair in the gut. In IBD, chronic inflammatory signals derail these pro-resolving functions, locking ISEMFs in a persistently activated state that favors fibrosis. Therapeutic strategies that enhance the pro-resolving and reparative programs of ISEMFs—rather than simply suppressing inflammation—may improve the quality of mucosal healing and reduce long-term fibrotic complications.

## 5 Therapeutic implications: targeting ISEMFs in IBD

### 5.1 Antifibrotic and immune-targeted therapies

Given the central role of intestinal subepithelial myofibroblasts (ISEMFs) in fibrogenesis, therapeutic strategies that modulate their activation, survival, or matrix-producing capacity represent a promising approach to preventing or treating fibrostenotic complications of inflammatory bowel disease (IBD). Traditional IBD therapies primarily target immune-mediated inflammation, which can indirectly slow fibrotic progression but generally fails to reverse established fibrosis. Clinical experience demonstrates that intestinal strictures may progress despite effective inflammatory control, underscoring the need for direct antifibrotic interventions.

One major antifibrotic strategy is inhibition of transforming growth factor-β (TGF-β) signaling, the dominant profibrotic pathway in intestinal fibroblasts. Pirfenidone, a small-molecule antifibrotic agent approved for idiopathic pulmonary fibrosis, suppresses TGF-β–driven collagen synthesis and fibroblast proliferation. In murine models of chronic colitis, pirfenidone significantly reduced intestinal collagen deposition and α-SMA–positive myofibroblast accumulation (38). Although clinical data in IBD remain limited, early pilot studies suggest pirfenidone may attenuate the progression of fibrostenotic Crohn’s disease, warranting further controlled trials.

Targeting downstream mediators of TGF-β is another approach. Connective tissue growth factor (CTGF) acts as a critical amplifier of fibrogenic signaling in stromal cells. CTGF inhibition has shown antifibrotic efficacy in multiple organs, and monoclonal antibodies such as pamrevlumab are in advanced clinical trials for pulmonary and pancreatic fibrosis (68). While not yet tested extensively in IBD, CTGF represents an attractive candidate target for intestinal fibrosis given its strong expression in fibrotic gut tissue.

In parallel, therapies that reshape the fibrogenic immune microenvironment indirectly reduce ISEMF activation. IL-23p19 inhibitors (e.g., risankizumab, guselkumab) effectively suppress Th17-driven inflammation and reduce IL-17–dependent stromal activation. While antifibrotic outcomes were not primary endpoints, imaging-based analyses from Crohn’s disease trials suggest reduced progression of bowel wall thickening in responders (69). In contrast, IL-13 neutralization (e.g., tralokinumab) has not demonstrated clinical efficacy in ulcerative colitis, suggesting redundancy or organ-specific differences in fibrotic drivers between gut and other tissues.

Janus kinase (JAK) inhibitors represent a particularly attractive class because they block multiple cytokine pathways relevant to fibroblast activation, including IL-6, IL-11, and interferons. *In vitro* studies using human intestinal fibroblasts have shown that tofacitinib suppresses cytokine-induced collagen synthesis and α-SMA expression, indicating a direct antifibrotic effect beyond immunosuppression (70). Clinically, long-term extension studies of ulcerative colitis patients treated with tofacitinib have not shown accelerated fibrotic complications, and anecdotal reports describe improvement of fistulizing disease, suggesting favorable stromal remodeling.

Anti-TNF therapies (e.g., infliximab, adalimumab) remain foundational treatments in IBD and exert secondary effects on ISEMFs. Beyond reducing inflammatory stimuli, TNF blockade has been shown to induce apoptosis of activated intestinal myofibroblasts and reduce mucosal α-SMA expression in responders (71). However, anti-TNF agents are generally insufficient to reverse established strictures, highlighting the limitation of anti-inflammatory therapy alone once fibrosis is advanced.

Combination strategies that simultaneously target inflammation and fibrosis are therefore of high interest. For example, angiotensin receptor blockers such as losartan, which downregulate TGF-β signaling, have shown antifibrotic effects in experimental colitis models and could theoretically complement biologic therapies (60). Although clinical evidence is preliminary, such combinations exemplify a rational approach to interrupting parallel inflammatory and fibrotic pathways.

**Figure 2:**
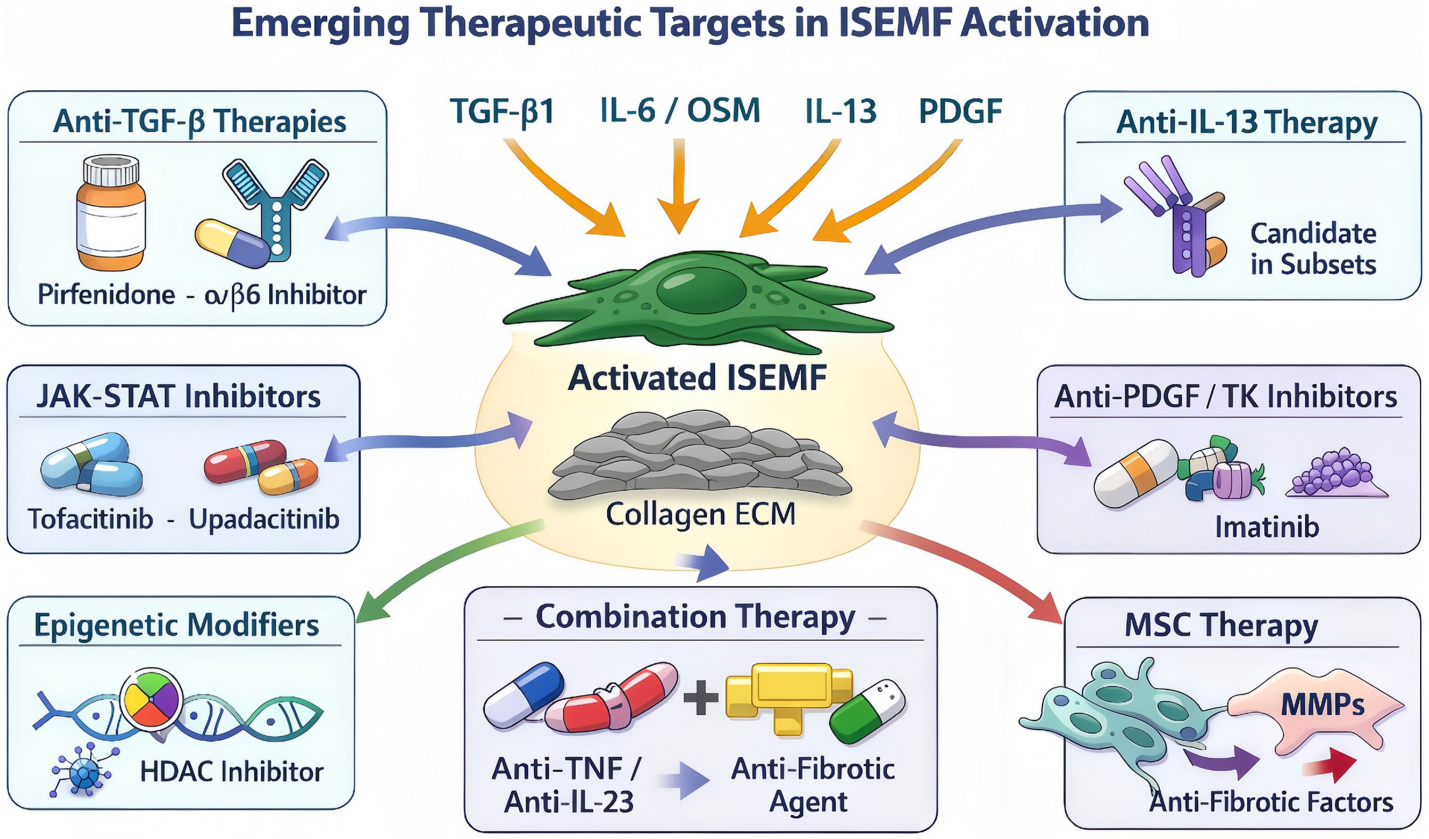
Emerging therapeutic strategies targeting ISEMF activation and fibrogenic signaling in IBD. Multiple profibrotic mediators—including TGF-β1, IL-6, IL-13, platelet-derived growth factor (PDGF), and oncostatin M (OSM)—converge on intestinal subepithelial myofibroblasts (ISEMFs) to promote myofibroblast differentiation, survival, and excessive extracellular matrix (ECM) deposition. Targeted therapeutic approaches aim to interrupt these pathways at different levels. Anti-TGF-β strategies (e.g., pirfenidone, integrin αvβ6 inhibitors) suppress core fibrogenic signaling, while JAK–STAT inhibitors (e.g., tofacitinib, upadacitinib) attenuate cytokine-driven activation downstream of IL-6, OSM, and IL-23. Additional approaches include anti-IL-13 therapies (with context-dependent efficacy), tyrosine kinase inhibitors targeting PDGF signaling (e.g., imatinib), and metabolic or epigenetic modulators (AMPK activators, HDAC inhibitors) designed to reprogram activated ISEMFs toward a quiescent phenotype. Combination strategies pairing immune suppression (e.g., anti-TNF or anti-IL-23) with fibrosis-directed agents, as well as regenerative therapies such as mesenchymal stromal cell (MSC) administration, represent rational avenues to prevent or reverse ISEMF-driven intestinal fibrosis.

Finally, microbiome-based therapies may modulate ISEMF behavior indirectly. Commensal-derived metabolites can restrain fibroblast activation, whereas dysbiosis promotes profibrotic signaling. Fecal microbiota transplantation (FMT) has demonstrated efficacy in inducing remission in ulcerative colitis and may reduce tissue injury and remodeling markers (72). While direct antifibrotic effects of FMT remain unproven, restoration of microbial homeostasis may create a permissive environment for ISEMF deactivation and scar resolution. Figure 2 illustrates the major profibrotic signals converging on ISEMFs and highlights current and emerging therapeutic strategies aimed at disrupting fibroblast activation, reprogramming fibrogenic pathways, and integrating anti-inflammatory and anti-fibrotic approaches for comprehensive IBD management.

### 5.2 Emerging and future therapies

The antifibrotic therapeutic pipeline in IBD is rapidly expanding, informed by advances in other fibrotic diseases. Several innovative strategies are under investigation. Recent single-cell and spatial studies suggest that fibroblast targeting in IBD should move beyond a generic “anti-fibroblast” strategy toward subset-informed intervention, because distinct stromal populations appear to differ in their localization, molecular programs, and contribution to fibrosis.

Among the fibroblast subsets currently implicated in intestinal fibrosis, the best defined are FAP^+^TWIST1^+^ fibroblasts, which were identified in fibrotic Crohn’s disease tissue by single-cell analysis as a major ECM-producing population enriched in stricturing lesions. These cells express FAP, TWIST1, COL1A1, POSTN, and other matrix-associated genes, and were shown to interact with CXCL9^+^ macrophages in a profibrotic stromal–myeloid niche (73). In addition, more inflammatory fibroblast states have been described in colitis and human IBD, including chemokine-enriched fibroblast programs expressing mediators such as IL6, IL33, CCL19, and CCL21, as well as spatially organized inflammatory fibroblasts that may support leukocyte recruitment and tissue remodeling (74, 75). More recent spatial work in stricturing Crohn’s disease also supports the coexistence of inflammatory fibroblasts and collagen-high fibroblasts, suggesting that fibrogenic stromal programs may be functionally partitioned across inflammatory and matrix-remodeling niches (76).

Proof-of-concept targeted intervention has already been reported for at least one such subset. In a Crohn’s disease fibrosis study, genetic deletion or pharmacologic inhibition of TWIST1 significantly attenuated intestinal fibrosis in mice, supporting the feasibility of targeting the FAP^+^TWIST1^+^ fibroblast program rather than fibroblasts indiscriminately (73).

TGF-β receptor kinase inhibitors, such as vactosertib, aim to selectively block TGF-β signaling at the receptor level. Preclinical studies demonstrate potent antifibrotic effects, but systemic toxicity remains a concern due to the pleiotropic roles of TGF-β in immune regulation and tumor suppression (77). Localized delivery approaches, including drug-eluting stents or endoscopic injection into strictures, are being explored to mitigate systemic risks.

Integrin-targeted therapies offer another means to limit fibroblast activation. Integrins such as αvβ6 and αvβ1 activate latent TGF-β stored in the extracellular matrix. Inhibition of αv integrins has shown antifibrotic effects in lung and liver models, and similar mechanisms may operate in the gut (78). Although still speculative in IBD, integrin-mediated TGF-β activation represents a compelling stromal-specific target.

Targeting ISEMF survival and apoptosis resistance is another emerging strategy. Pathogenic myofibroblasts exhibit upregulation of anti-apoptotic pathways, allowing them to persist after injury resolution. Pharmacologic induction of fibroblast apoptosis—using agents that disrupt BCL-2 or PI3K/AKT signaling—has shown promise in experimental fibrosis models (79). Translation to IBD will require gut-selective delivery to avoid systemic toxicity.

Emerging delivery-based strategies may further improve stromal selectivity. Although most remain preclinical, RNA-based and nanomedicine-enabled approaches are increasingly being explored in intestinal fibrosis and IBD. For example, miR-29a-loaded nanoparticles reduced inflammation and fibrosis in murine colitis/fibrosis models and also suppressed TGF-β1-driven profibrotic responses in fibroblast-like cells, providing a proof-of-concept example for RNA-based antifibrotic delivery (80). More broadly, current reviews of intestinal fibrosis therapeutics suggest that nanocarrier systems may help overcome the delivery barriers that have limited local antifibrotic treatment in the gut, although true fibroblast-subset targeting remains at an early stage (81, 82).

Finally, personalized antifibrotic therapy is emerging as a key concept. Biomarkers of active fibrogenesis—such as circulating collagen neoepitopes (e.g., PRO-C3)—and genetic risk variants may help identify patients at high risk of fibrostenotic progression who would benefit from early antifibrotic intervention (83). Ultimately, combination regimens pairing biologics with antifibrotic agents may become standard for patients with high fibrotic risk.

In summary, targeting ISEMFs represents a paradigm shift in IBD management—from reactive treatment of established strictures to proactive prevention of fibrosis. As antifibrotic strategies mature, future IBD care may increasingly incorporate early intervention against fibrogenesis, to make surgery for fibrostenotic disease a rarity rather than an inevitability.

An expanding array of therapeutic strategies is now being explored to modulate ISEMF activation and fibrogenesis in IBD, ranging from immunomodulatory biologics and small-molecule inhibitors to antifibrotic agents, stem cell–based approaches, and microbiome-targeted interventions. Table 2 summarizes current and emerging therapies that directly or indirectly affect ISEMF biology, their proposed mechanisms of action, and their stage of clinical development.

**Table 2:** Current and emerging therapies targeting intestinal subepithelial myofibroblasts and fibrogenesis in inflammatory bowel disease.

Therapeutic class/pathway	Example agents	Proposed mechanism relevant to ISEMFs/fibrosis	Evidence base (selected PubMed-indexed examples)	Clinical status in IBD	Key limitations/notes	Reference
TGF-β pathway modulation (direct/indirect)	Anti–TGF-β strategies (conceptual); SMAD7 antisense (mongersen); upstream activation blockade (integrin-mediated TGF-β activation: concept)	Reduce SMAD-driven collagen transcription; re-balance ECM turnover and myofibroblast differentiation programs.	Mongersen improved clinical outcomes in phase 2 Crohn’s (proof-of-concept for gut-targeted TGF-β pathway modulation).	Mongersen program discontinued after later-stage failure; TGF-β targeting remains an active concept.	Systemic TGF-β blockade risks immune dysregulation; localized delivery is critical.	D’Haens et al. (96)
Broad anti-fibrotic small molecules	Pirfenidone	Inhibits fibroblast proliferation and TGF-β signaling; reduces collagen production and myofibroblast features.	Oral pirfenidone reduced fibrosis in a murine colitis model and inhibited TGF-β signaling.	Not approved for IBD fibrosis; off-label interest/preclinical rationale.	Translational gap: endpoints for IBD strictures, dosing, and long-term safety in IBD populations.	Ferrante et al. (97)
IL-23 pathway inhibition (Th17 axis upstream)	Risankizumab (p19); other IL-23p19 agents	Decreases IL-23–driven inflammatory circuits that sustain stromal activation; may indirectly reduce profibrotic signaling in the mucosa.	Phase 3 induction (ADVANCE/MOTIVATE) and maintenance (FORTIFY) trials demonstrate efficacy in Crohn’s (inflammation control), enabling assessment of longer-term complication modification.	Approved/advanced for Crohn’s; fibrosis-specific endpoints still evolving.	Anti-fibrotic benefit likely indirect; stricturing cohorts and imaging endpoints needed.	Feagan et al. (69)
IL-17A blockade (downstream Th17)	Secukinumab	Theoretical reduction in IL-17–driven stromal activation; however IL-17 supports barrier defense.	Randomized trial in Crohn’s showed unexpected lack of benefit and safety concerns, highlighting IL-17’s protective roles in gut.	Not used for Crohn’s; generally avoided.	Demonstrates that “anti-fibrotic by cytokine neutralization” can backfire if barrier immunity is compromised.	Hueber et al. (98)
JAK inhibitors (multi-cytokine signal blockade)	Tofacitinib (pan-JAK; UC), Upadacitinib (JAK1; Crohn’s)	Suppresses signaling downstream of multiple cytokines relevant to stromal activation (IL-6-family, OSM/IL-11; interferons), potentially reducing chemokine programs and survival of activated myofibroblasts.	NEJM UC trials show tofacitinib efficacy; NEJM Crohn’s trials show upadacitinib induction/maintenance benefit—both support durable inflammation suppression and potential secondary impact on fibrostenosis risk.	Tofacitinib approved for UC; upadacitinib approved/late-stage for Crohn’s (region-dependent).	Long-term effects on strictures/fibrosis require dedicated endpoints; safety monitoring (infection, thrombosis risk) is essential.	Monteleone et al. (99)
Anti-TNF agents (immune + direct stromal effects)	Infliximab, adalimumab	Reduce inflammatory drive; may also directly alter myofibroblast behavior (MMP/TIMP balance, migration, apoptosis susceptibility).	Anti-TNF antibodies can functionally modulate Crohn’s disease myofibroblasts ex vivo/in vitro.	Standard of care for IBD inflammation; limited for established strictures.	Often insufficient once fibrosis is advanced; may need combination with anti-fibrotics.	Li et al. (100)
Stromal inflammatory module targeting	OSM–OSMR axis (conceptual), downstream blockade (e.g., JAK/STAT approaches)	Reduce stromal chemokine/adhesion programs that sustain leukocyte retention and chronic activation.	OSM abundance predicts anti-TNF non-response; OSM drives a stromal inflammatory state in IBD.	Not yet standard; target validation supports drug development interest.	Requires careful patient stratification (biomarker-guided therapy).	Costello et al. (72)
Cell therapy (immunomodulatory + pro-repair secretome)	Darvadstrocel (Cx601) for perianal Crohn’s; other MSC products under study	MSC secretome may reduce inflammatory cytokines and influence matrix remodeling; potential indirect effects on activated stromal cells in fistula tracts.	Phase 3 trial showed Cx601 efficacy for complex perianal fistulas in Crohn’s.	Approved in some regions for perianal fistulizing Crohn’s.	Luminal fibrosis/stricture impact unclear; engraftment vs. paracrine effects remain debated.	Paramsothy et al. 2017 (62)
Microbiome-based interventions	FMT (UC); diet/high-fiber strategies (adjunct)	Modify luminal ecology → increase beneficial metabolites and reduce pro-inflammatory triggers that sustain stromal activation.	Randomized trials show FMT can induce UC remission (short-term), supporting a tractable lever on mucosal inflammatory tone.	Investigational/adjunct; protocols heterogeneous.	Standardization (donor, dosing, delivery) and durability remain key challenges.	Panés et al. (67)

This table outlines therapeutic strategies that directly or indirectly modulate intestinal subepithelial myofibroblast (ISEMF) activation, survival, and extracellular matrix production in inflammatory bowel disease (IBD). Listed therapies include approved biologics and small-molecule inhibitors, antifibrotic agents repurposed from other organ systems, stem cell–based approaches, and microbiome-directed interventions. Mechanisms of action, clinical development status, and key limitations relevant to fibrotic disease modification are highlighted.

## 6 Challenges and future directions

### 6.1 Unresolved mechanisms and knowledge gaps

Despite major advances in understanding intestinal subepithelial myofibroblast (ISEMF) biology, several fundamental questions remain unresolved and represent critical barriers to effective antifibrotic therapy in inflammatory bowel disease (IBD).

### 6.2 Triggers of persistent ISEMF activation

A central unanswered question is why ISEMFs in certain patients transition into a persistently activated, fibrogenic state, whereas others with comparable inflammatory burden do not develop progressive fibrosis. Genetic susceptibility likely contributes to this divergence. Variants in genes associated with Crohn’s disease risk—such as NOD2, ATG16L1, and components of extracellular matrix turnover pathways—may influence fibroblast responses to inflammatory or microbial cues (84). Environmental factors also modulate fibrotic risk; for example, cigarette smoking is strongly associated with fibrostenotic Crohn’s disease and may enhance mesenchymal activation through oxidative stress and altered immune signaling.

At a mechanistic level, it remains unclear whether there is a definable “point of no return” at which ISEMFs become irreversibly programmed toward fibrosis. Emerging evidence suggests that chronic inflammation induces stable epigenetic changes in fibroblasts, including altered DNA methylation and histone modifications at profibrotic loci, which may lock cells into a myofibroblast phenotype even after inflammation subsides (58). Longitudinal studies using serial biopsies from early disease through established strictures are urgently needed to define the temporal sequence of molecular events driving this transition.

### 6.3 ISEMF heterogeneity and functional specialization

Single-cell transcriptomic analyses have overturned the notion that intestinal fibroblasts represent a homogeneous population. Multiple fibroblast subsets have now been described in human IBD, including inflammatory fibroblasts that secrete chemokines, extracellular matrix–producing fibrogenic fibroblasts enriched for collagen and fibroblast activation protein (FAP), and myogenic fibroblasts expressing smooth muscle–related genes (85, 86). However, the lineage relationships and plasticity among these populations remain poorly defined.

A key unresolved issue is whether inflammatory fibroblasts can transdifferentiate into fibrogenic fibroblasts under sustained inflammatory pressure, or whether these subsets arise from distinct progenitors. Clarifying this distinction is critical for therapy design, as targeting a single pathogenic fibroblast subset—such as FAP^+^ collagen-producing fibroblasts—may spare fibroblast populations required for normal repair. Spatial transcriptomics and multiplex imaging approaches applied to human IBD tissue are beginning to map fibroblast subsets within specific anatomic niches (mucosa, submucosa, muscularis propria), but further work is required to link spatial location to functional outcome.

### 6.4 Immune–stromal crosstalk

Although it is well established that ISEMFs engage in bidirectional communication with immune cells, the precise dynamics of these interactions during chronic inflammation remain incompletely understood. Th17-associated cytokines such as IL-17A and IL-22 exemplify this complexity. IL-22 promotes epithelial regeneration during acute injury, yet persistent IL-22 signaling has been linked to stromal activation and fibrosis in chronic inflammatory settings (87). Determining how timing, magnitude, and cellular context dictate these divergent outcomes is a major challenge.

Recent studies have identified spatially organized immune–stromal niches in fibrotic Crohn’s disease, including aggregates of CXCL9^+^ macrophages co-localizing with FAP^+^ fibroblasts, suggesting the existence of self-reinforcing chemokine circuits that sustain fibrosis (88). Disrupting such axes—for example, by targeting CXCL9/CXCR3 signaling—could represent a novel antifibrotic strategy, but careful evaluation is needed to avoid impairing host defense and immune surveillance.

### 6.5 Differences between Crohn’s disease and ulcerative colitis fibrosis

Fibrosis is far more prevalent and clinically significant in Crohn’s disease than in ulcerative colitis, yet the mechanistic basis for this difference remains unclear. While transmural inflammation and deeper mesenchymal involvement in Crohn’s disease undoubtedly contribute, emerging data suggest intrinsic differences in fibroblast programming between the two conditions. Comparative studies indicate that fibroblasts from ulcerative colitis tissue may preferentially adopt an inflammatory, cytokine-secreting phenotype, whereas Crohn’s-derived fibroblasts are more prone to collagen production and contractility (89). Elucidating disease-specific fibroblast signatures may explain differential responses to therapy and guide disease-tailored antifibrotic interventions.

### 6.6 Epigenetic regulation and microRNAs

Stable epigenetic alterations are increasingly recognized as key drivers of persistent fibrosis. Differential DNA methylation and chromatin accessibility at collagen and cytoskeletal gene loci have been reported in fibroblasts isolated from fibrotic intestinal tissue (83). Histone-modifying enzymes, including specific histone deacetylases (HDACs), may therefore represent therapeutic targets to “reset” fibroblast gene expression programs.

MicroRNAs also play a central role in fibrotic regulation. The miR-29 family, a well-established suppressor of collagen gene expression, is consistently downregulated in fibrotic tissues across organs. Experimental restoration of miR-29 reduces collagen synthesis in lung and liver fibrosis models, raising the possibility that similar approaches could be applied to intestinal fibroblasts (90). The major challenge remains safe and efficient delivery of epigenetic or RNA-based therapies to stromal cells *in vivo*.

Intestinal fibrosis in IBD represents a maladaptive consequence of dysregulated tissue repair, with ISEMFs occupying a central pathogenic role. Although modern biologic and small-molecule therapies have transformed inflammatory control, their impact on fibrotic progression has been limited. Insights into ISEMF heterogeneity, immune–stromal crosstalk, and epigenetic reprogramming now provide an opportunity to move beyond symptom control toward mechanism-based antifibrotic intervention. Future research priorities include:

## 7 Biomarkers and early detection

Development of non-invasive biomarkers of active fibrogenesis—such as serum collagen neo-epitopes (e.g., PRO-C3) or advanced imaging markers—will be essential for identifying patients at risk before irreversible strictures develop (91).

## 8 Refined therapeutic targeting

Identification of fibroblast-specific drivers, such as transcriptional regulators or metabolic pathways uniquely active in pathogenic ISEMF subsets, may enable selective antifibrotic therapy without impairing normal wound healing.

## 9 Advanced preclinical models

Improved experimental systems—including chronic colitis models that reliably develop fibrosis and human organoid-stromal co-culture platforms—are needed to test antifibrotic strategies under conditions that more closely resemble human disease (92).

## 10 Personalized medicine approaches

Integrating genetic risk, molecular fibroblast signatures, and clinical phenotype may allow stratification of patients for early antifibrotic intervention, ushering in a precision medicine approach to fibrostenotic IBD.

## 11 Conclusion

This review highlights the central role of intestinal subepithelial myofibroblasts (ISEMFs) at the intersection of inflammation, tissue repair, and fibrosis in inflammatory bowel disease (IBD). Once regarded primarily as passive structural elements, ISEMFs are now recognized as dynamic and multifunctional stromal cells that actively shape the intestinal microenvironment. Under physiological conditions, they support epithelial renewal, immune homeostasis, and wound resolution. In the context of chronic intestinal inflammation, however, these same reparative programs are subverted, leading to persistent myofibroblast activation, excessive extracellular matrix deposition, and progressive fibrosis.

We have summarized how sustained exposure to inflammatory and microbial signals in IBD drives pathological ISEMF reprogramming through key profibrotic pathways, including TGF-β/Smad, IL-6/JAK-STAT3, and related signaling networks. Crosstalk between ISEMFs and immune cells—particularly Th17 cells, macrophages, and neutrophils—establishes self-reinforcing inflammatory–fibrotic circuits that can persist even after mucosal inflammation appears clinically controlled. These mechanisms help explain why intestinal fibrosis often progresses independently of overt inflammatory activity and why current anti-inflammatory therapies alone are insufficient to prevent fibrostenotic complications (18).

A recurring theme throughout this review is stromal heterogeneity. Emerging single-cell and spatial transcriptomic studies demonstrate that ISEMFs comprise multiple phenotypically and functionally distinct subsets, some of which may promote inflammation, others fibrosis, and still others tissue repair. Dissecting the lineage relationships and plasticity among these subsets will be essential for developing targeted therapies that selectively suppress pathogenic fibroblast populations while preserving those required for normal healing (85). The recognition that bone marrow–derived cells and immune cell transdifferentiation may contribute to the fibroblast pool further underscores the systemic nature of fibrogenesis in IBD.

From a therapeutic perspective, it is increasingly clear that multimodal strategies will be required to address intestinal fibrosis effectively. Future progress will depend on several parallel advances: (1) development of sensitive biomarkers and imaging tools to detect active fibrogenesis before irreversible scarring occurs; (2) rigorous clinical testing of antifibrotic agents—many repurposed from other fibrotic diseases—in well-defined IBD populations enriched for stricturing risk; (3) rational combination therapies that simultaneously suppress inflammation and interrupt fibroblast activation; and (4) adoption of precision medicine approaches that integrate genetic, transcriptomic, and microbiome data to stratify fibrotic risk and guide individualized treatment.

In closing, while control of inflammation remains the cornerstone of IBD management, preventing and treating fibrosis represents the next critical frontier. Fibrosis is no longer viewed as an inevitable end stage of disease, but as a dynamic and potentially modifiable process. The growing body of knowledge on ISEMF biology provides a roadmap for this paradigm shift. By translating mechanistic insights into targeted clinical interventions, the field moves closer to a future in which remission in IBD signifies not only the absence of inflammation, but also preservation—or restoration—of normal intestinal architecture and function. With continued interdisciplinary efforts bridging basic stromal biology, translational research, and clinical trials, there is justified optimism that fibrotic complications of IBD can ultimately be prevented or even reversed.

## Glossary

α-SMA: Alpha-smooth muscle actin
AMPK: AMP-activated protein kinase
ATG16L1: Autophagy related 16 like 1
CAF: Cancer-associated fibroblast
CCL: C-C motif chemokine ligand
CCR: C-C motif chemokine receptor
CD: Crohn’s disease
CTGF: Connective tissue growth factor
CXCL: C-X-C motif chemokine ligand
CXCR: C-X-C motif chemokine receptor
DSS: Dextran sulfate sodium
ECM: Extracellular matrix
EGF: Epidermal growth factor
EGFR: Epidermal growth factor receptor
FAK: Focal adhesion kinase
FAP: Fibroblast activation protein
FOXL1: Forkhead box L1
FSP1: Fibroblast-specific protein 1
HGF: Hepatocyte growth factor
HSCT: Hematopoietic stem cell transplantation
IBD: Inflammatory bowel disease
IFN: Interferon
IGF-1: Insulin-like growth factor 1
IL: Interleukin
IPA: Indole-3-propionic acid
ISEMFs: Intestinal subepithelial myofibroblasts
JAK: Janus kinase
Lgr5: Leucine-rich repeat-containing G-protein coupled receptor 5
MHC: Major histocompatibility complex
MMP: Matrix metalloproteinase
MMT: Macrophage-to-myofibroblast transition
MSC: Mesenchymal stem/stromal cell
NETs: Neutrophil extracellular traps
NF-κB: Nuclear factor kappa B
NOD2: Nucleotide-binding oligomerization domain-containing protein 2
OSM: Oncostatin M
OSMR: Oncostatin M receptor
PDGF: Platelet-derived growth factor
PDGFRA: Platelet-derived growth factor receptor alpha
PPAR: Peroxisome proliferator-activated receptor
PRO-C3: Pro-collagen type III neo-epitope
PXR: Pregnane X receptor
ROS: Reactive oxygen species
STAT: Signal transducer and activator of transcription
TGF-β: Transforming growth factor beta
TIMP: Tissue inhibitor of metalloproteinase
TLR: Toll-like receptor
TNBS: Trinitrobenzene sulfonic acid
TNF-α: Tumor necrosis factor alpha
UC: Ulcerative colitis
VCAM-1: Vascular cell adhesion molecule 1
VEGF: Vascular endothelial growth factor
Wnt: Wingless-related integration site
YAP/TAZ: Yes-associated protein / transcriptional coactivator with PDZ-binding motif
